# Puerperal women's social interactions related to their sexual health after childbirth

**DOI:** 10.1590/1980-220X-REEUSP-2023-0056en

**Published:** 2024-02-09

**Authors:** Wellery Stefany Nunes Glavina, Carla Marins Silva, Elaine Lutz Martins, Juliana Cristina dos Santos Monteiro, Isabelle Wengler Silva, Ana Paula Almeida Brito, Fernanda Marçal Ferreira, Ludmila de Oliveira Ruela, Raquel Gomes de Oliveira Tomaz

**Affiliations:** 1 Universidade de São Paulo, Escola de Enfermagem, São Paulo, SP, Brazil.; 2 Universidade de São Paulo, Escola de Enfermagem, Departamento de Enfermagem Materno-Infantil e Psiquiátrica, São Paulo, SP, Brazil.; 3 Universidade do Estado do Rio de Janeiro, Faculdade de Enfermagem, Departamento de Enfermagem Materno-Infantil, Rio de Janeiro, RJ, Brazil.; 4 Universidade de São Paulo, Escola de Enfermagem de Ribeirão Preto, Departamento de Enfermagem Materno-Infantil e Saúde Pública, Ribeirão Preto, SP, Brazil.; 5 Universidade de São Paulo, Hospital Universitário, São Paulo, SP, Brazil.; 6 Universidade de São Paulo, Escola de Enfermagem de Ribeirão Preto, Ribeirão Preto, SP, Brazil.

**Keywords:** Sexual health, Postpartum period, Women’s health, Health education, Obstetric nursing, Salud sexual, Periodo Posparto, Salud de la mujer, Educación en Salud, Enfermería obstétrica, Saúde sexual, Período pós-parto, Saúde da mulher, Educação em saúde, Enfermagem obstétrica

## Abstract

**Objective:**

To analyze puerperal women’s experiences of sexual health after childbirth from the perspective of symbolic interactionism.

**Method:**

Descriptive, qualitative study. Twenty semi-structured interviews were conducted by videoconference with women in the remote puerperium, captured by snowball technique and searched for “seeds” on Instagram®. Bardin’s content analysis and Symbolic Interactionism were used as references.

**Results:**

The puerperal women signify sexual health from a perspective of comprehensive healthcare. However, due to the duality between “being a woman” and “being a mother”, they recognize fear, bodily transformations and changes in focus from the love relationship to caring for the baby as factors that interfere with sexual health. And they choose to put themselves aside, prioritizing caring for others. They re-signify sexual health by recognizing the importance of taking care of themselves in biopsychosocial aspects and try to recover self-care for a healthy sexual experience.

**Conclusion:**

Despite the meanings attributed, women’s social interactions with the puerperium interfere negatively with sexual health. Professionals should be sensitized to the inclusion of actions that promote changes in the social action of these women towards self-care.

## RESUMO

**Objetivo:**

Analisar experiências de puérperas sobre saúde sexual após o parto na perspectiva do interacionismo simbólico.

**Método:**

Estudo descritivo, qualitativo. Realizadas 20 entrevistas semiestruturadas por videoconferência com mulheres no puerpério remoto, captação por “snowball” e busca das “sementes” pelo Instagram®. Utilizou-se a análise de conteúdo de Bardin e o Interacionismo Simbólico como referencial.

**Resultados:**

As puérperas significam saúde sexual numa perspectiva de integralidade dos cuidados em saúde. Contudo, pela dualidade entre o “ser mulher” e o “ser mãe”, reconhecem o medo, as transformações corporais e as mudanças de foco da relação amorosa para os cuidados com o bebê como fatores que interferem na saúde sexual. E, escolhem se deixar de lado, priorizando o cuidado com os outros. Ressignificam saúde sexual pelo reconhecimento da importância de cuidar de si nos aspectos biopsicossociais e tentam resgatar o autocuidado para vivência sexual saudável.

**Conclusão:**

Apesar dos significados atribuídos, as interações sociais das mulheres com o puerpério interferem negativamente na saúde sexual. Profissionais devem ser sensibilizados quanto à inclusão de ações que promovam mudanças da ação social dessas mulheres para o cuidado de si.

## RESUMEN

**Objetivo:**

Analizar las experiencias de salud sexual de las puérperas después del parto desde la perspectiva del interaccionismo simbólico.

**Método:**

Estudio descriptivo y cualitativo. Se realizaron 20 entrevistas semiestructuradas por videoconferencia con mujeres puérperas a distancia, captadas mediante bola de nieve y búsqueda de las “semillas” a través de Instagram®. Se utilizaron como referencias el análisis de contenido de Bardin y el Interaccionismo Simbólico.

**Resultados:**

Las puérperas significan la salud sexual desde una perspectiva de atención sanitaria integral. Sin embargo, debido a la dualidad entre “ser mujer” y “ser madre”, reconocen el miedo, las transformaciones corporales y los cambios de enfoque de la relación amorosa al cuidado del bebé como factores que interfieren en la salud sexual. Y deciden dejarse a un lado, dando prioridad al cuidado de los demás. Resignifican la salud sexual reconociendo la importancia de cuidarse en los aspectos biopsicosociales e intentan recuperar el autocuidado para una experiencia sexual saludable.

**Conclusión:**

A pesar de los significados atribuidos, las interacciones sociales de las mujeres con el puerperio interfieren negativamente en la salud sexual. Los profesionales deben ser sensibilizados para la inclusión de acciones que promuevan cambios en las acciones sociales de estas mujeres hacia el autocuidado.

## INTRODUCTION

Sexual health refers to the state of physical, emotional, mental and social well-being related to sexuality. In that sense, for individuals to achieve satisfactory sexual health, there is a need for access to comprehensive information, quality health care and an environment that defends and promotes sexual health, alongside the possibility of having safe and pleasurable sexual experiences, without coercion, discrimination and violence^(1,2)^, including in phases such as the postpartum period.

The puerperium physiologically comprises the involutionary and recovery processes of the maternal organism after pregnancy, also characterized by marked changes in other aspects of women’s lives, be they anatomical, physiological, endocrine, socio-cultural, emotional, marital, family, social or professional^(2)^. These multifactorial changes can affect women’s well-being and relationships.

Adaptations to new demands, physical and hormonal changes, added to cultural and emotional issues and a lack of knowledge on the subject, delay the resumption of sexual activities and contribute to a decrease in the frequency of consummated sexual relations due to low sexual desire and low sexual satisfaction among women and their partners. Furthermore, these adaptations can lead to difficulties in harmonizing the couple’s intimate life with caring for the baby^(3,4)^. They can also be a source of discomfort, insecurity, conflict and distancing from the partner^(5)^.

In the puerperium, women’s sexual health is often delegitimized and neglected in the face of the demands of motherhood and caring for the baby, so sexual intercourse may not be motivated by the satisfaction of personal pleasure, but rather the pleasure of the partner, out of marital duty or to avoid infidelity^(6,7)^. In addition, women after childbirth are more vulnerable to sexual dysfunctions, such as problems with lubrication, libido, arousal, orgasm and dyspareunia, mainly due to a hormonal drop in estrogen and progesterone, and the production of prolactin, hormones that interfere with sexuality^(8)^, as well as self-image and self-esteem disorders in relation to their bodies^(5,9)^. Thus, there is a need to take a broader look at the care provided to these women.

Studies have highlighted the challenges of returning to sexual activity^(5,6,8)^ and the interface between breastfeeding and sexuality^(4)^. Therefore, there is a need to broaden reflections on all issues involving sexual health in the postpartum period, including by health professionals, with a view to reducing sexual disorders^(3,6,7)^.

Against this backdrop, the guiding questions of this research were: what meanings do puerperal women attribute to sexual health? What is the experience of puerperal women in relation to their sexual health after childbirth?

In line with the interactionist perspective, considering the understanding of meanings and process from the perspective of the other, the aim of this study was to analyze the experiences of puerperal women about sexual health after childbirth from the perspective of symbolic interactionism.

## METHOD

### Theoretical Framework

Symbolic Interactionism (SI) was adopted as the theoretical framework used to observe human behavior. According to this perspective, people construct meanings from their interactions with each other and these meanings are incorporated into objects, institutions and social norms^(10)^. According to SI, due to the load of meanings in the different situations experienced by individuals, reality is constructed through a constant negotiation of meanings between individuals in social interactions. This symbolic interaction shapes future perceptions and behavior and is, in turn, influenced by the cultural and social expectations present in society^(11)^.

The SI is based on three premises which, in relation to the object of this study, are as follows: puerperal women act in relation to sexual health according to the meanings it has for them; the meanings of sexual health arise from the social interaction they establish with others; they can modify the meanings they attribute to sexual health by interpreting the things and situations they encounter in this context. Thus, based on this interaction, they can re-signify and draw up lines of action in relation to this issue. For this analysis, a representative scheme of human action was used from the interactionist perspective proposed by Charon^(10)^.

### Type of Study

This is a descriptive study with a qualitative approach, focusing on analyzing the human expressions present in relationships, subjects and representations^(12)^.

### Site, Population and Selection Criteria

Conducted in a virtual environment, the inclusion criteria were: Brazilian women, 42 days to one year postpartum, regardless of the route of birth, whether normal or cesarean, aged 18 or over. We excluded women who had gestational complications, complicated births, stillbirths or mothers of children with any disability or illness; women who had speech impairments, as well as disorders that made it impossible to collect data and who did not have an e-mail address or who did not have access to the internet or a videoconferencing application.

Sampling took place using the snowball technique, in which initial participants (seeds) with the necessary profile for data collection are located in the general population. These “seeds” help the researcher to start making contacts and feel out the group to be studied, indicating other people with the desired profile. This process takes place successively until the “saturation point” is reached^(13)^. To capture the “seed” participants, the online social network Instagram® was used, given its wide audience reach and the possibility of creating visually attractive posts that clearly explained the purpose of the research. In this way, the chance of participation by the target audience was increased, people from different locations were attracted and the research theme was made more visible. Initially, a post was created with the objectives and the inclusion and exclusion criteria for taking part in the study. When the first five candidates for taking part in the study expressed an interest in contributing, the researcher contacted them individually by private message, still on Instagram®. In this exchange of messages, the Informed Consent Form was sent, the reasons for interest in the research topic were reported and a subsequent online meeting was scheduled for the videoconference interview, recorded using the Google Meet® application, a free communication service. The interview was conducted online, allowing access to women from different locations across the country. It was recommended that the participant choose a private location so that her privacy was maintained during data collection. Each seed participant indicated other possible participants and so on. The saturation point occurred due to the volume of data and the degree of detail and depth, demonstrating the various dimensions of the research object.

### Data Collection

A semi-structured interview script was used, consisting of two parts. The first consisted of objective characterization questions, such as: age; marital status; level of education; state; municipality; family income; and length of time postpartum. The second part included a trigger question: “What does sexual health mean to you in the postpartum period?”, and topics introduced during the interview to make the questions more flexible and in-depth. The instrument was pilot-tested and no changes were needed.

The sound and image of the interview were recorded using Google Meet® for transcription and data analysis. The data was collected between March and June 2022, at times that suited the participants’ availability, and the interviews lasted an average of twenty-nine minutes. The interviews were conducted by an obstetric nursing resident, the author of this manuscript, who was trained in the data collection strategy adopted. The participants were protected in case they wanted to review any information on the recording, and could keep a copy of the file, but none of them requested it.

### Data Analysis

The data obtained was analyzed using Atlas.ti9 software, according to Bardin’s (14) content analysis assumptions. The software was used to select excerpts of speech (*quotations*), create codes and group codes (*groups*). Content analysis was organized into three stages: pre-analysis; exploration of the material; and treatment, with inference and interpretation of the results^(14,15)^.

### Ethical Aspects

All the ethical requirements of the National Health Council were respected, in accordance with resolutions 466/2012 and 510/2016^(16,17)^. The project was cleared under opinion number 5.266.113, in February 2022, by the Research Ethics Committee of the School of Nursing of the University of São Paulo. A Free and Informed Consent Form (FICF) was used for all participants. The statements were identified by the letter E followed by the interview number, for example, E3.

## RESULTS

Using the snowball sampling technique, 47 women expressed an interest in taking part in the study. Of these, five women booked the interview online and didn’t show up on the scheduled date. In addition, one woman began the interview but did not feel comfortable answering the questions and was therefore excluded from the sample. Two pilot tests were carried out, but due to the richness of the content of these interviews, they were included in the total to be analyzed. Thus, 20 women took part, residents of the state of São Paulo, aged between 19 and 42, with an average age of 30, mostly married (n = 9, 45.0%), with a family income of between one and three minimum wages (n = 9, 45%) and with completed high school (n = 11, 55.0%). The participants were predominantly four months postpartum (n = 6, 30.0%), with a prevalence of care provided by the Unified Health System (n = 15, 75.0%) and had attended an average of nine antenatal appointments and two postpartum appointments.

### Understanding Sexual Health in a Broader Sense

From the social interaction that the women established with others, added to past memories and the baggage of knowledge built up by formal education, the media, professional guidance or guidance from people close to them, the participants attributed meanings to sexual health as something beyond the sexual act, from a perspective of comprehensive health care.

For them, sexual health involves aspects of mental health, care and self-knowledge. In the sense of taking care of themselves and others, as well as knowing the limits of their own bodies.

*I think it speaks a lot about women’s holistic health, not just the sexual act itself, but also mental health. (E19)*

*I think it’s knowing the limits you have in a relationship and also with your body. (E5)*

From a physical and biological care perspective, women mean sexual health as prevention of sexually transmitted infections (STIs) and other diseases, medical consultations and regular examinations.

*Sexual health includes contraception, so as not to acquire diseases. (E14)*

*For me, it’s both of us going to the doctor all the time. Doing the pap [cytopathology test] and the routine exams all correctly. (E3)*

Another meaning attributed to sexual health was a healthy relationship between the couple. For them, it’s about quality sexual intercourse that is pleasurable for both, absence of discrimination and violence, with dialog and respect, having the freedom to do or not do what they like without impositions.

*Then there’s libido, isn’t there, there’s desire on both sides, it’s reciprocal and pleasurable for both sides. (E9)*

*It’s about having a healthy life, a person who doesn’t suffer any kind of discrimination or violence. (E1)*

### Recognizing Factors that Negatively Interfere with their Postpartum Sexual Health

Defining the situation for themselves, in the interpretative process used by women when relating to the situations they find themselves in, they reflect on their postpartum period and recognize that it is a period marked by the duality between “being a woman” and “being a mother”, with changes in social roles, emotional vulnerability and changes in their routine with an accumulation of functions.

*Because when you become a mother, you forget your woman side a bit, don’t you, so I forgot my woman side a bit, you forget that you’re a woman and you become a mother. (E17)*

*Once you’re a mother, you have to be like an octopus, don’t you, divided into a thousand, between husband, child and tasks in general. (E2)*

In the early stages of the puerperium, they faced challenges in adapting to this new condition. The changes involve restructuring their lives and the lives of the couple. And they reported the loss and mourning of life before the birth, with thoughts that their lives would never return to the way they were.

*We think that life will never return to normal, it’s such a whirlwind of feelings. I just cried because I thought life was over for me, as if it felt like I’d lost the life I had before. (E1)*

*I’ll never be what I was before I had a child, that person died in childbirth. (E6)*

In this challenging postpartum context, for the participants, fear is a factor that impacts on postpartum sexual health, including: fear of returning to sexual activities, fear due to the cesarean section or perineal stitches and fear of a new pregnancy.

*There’s that fear, isn’t there, of having gone through a caesarean section, that fear of something happening. (E1)*

*After the postpartum, my biggest fear was getting pregnant again. So that never left my mind, I was scared to death of becoming pregnant in the postpartum period. (E16)*

Bodily transformations after childbirth are also factors that negatively affect sexual health. They report that, before, their body was seen as an erotic body, now a maternal body, a sanctuary dedicated entirely to their child. As a result, there are changes in their self-image and self-esteem, expressing dissatisfaction with their body and a feeling of being physically unattractive. As a result, they recognize that they don’t think about their sexual health in the puerperium. They report a change in their willingness to engage in sexual activity and a reduction in libido in the postpartum period.

*I no longer feel comfortable being undressed. It’s not the same, sexually speaking, because I used to love the light on, now I don’t like it anymore [...] And the breasts? they’re my son’s food, like, I used to like looking at them, but it’s not a part of my body that I think is cool anymore. (E9)*

*To this day I’m not 100% back, and that’s something that messes with my head and my relationship too. I confess, I don’t feel 100% comfortable with my body. (E14)*

*So, it killed my libido (laughs). I think it’s almost gone to zero, I rarely feel like it (E5).*

*My thirst, the desire has only diminished now that I’ve become a mother. It’s as if my libido had stayed in hospital. (E6)*

*There’s no need, there’s no need, it’s crazy. It’s something that has no space.*

Another factor that influences sexual health is the shift in focus from the love relationship to caring for the baby. The participants report significant negative impacts on marital relations, with reduced time with their partner and the presence of the baby, depriving the couple of their freedom.

*Now, especially after the second child, there is practically no time for him. (E6)*

*I think if I had a room just for the two of us, even with the tiredness, it would be easier, wouldn’t it, but with a child in the room it’s too bad, it’s horrible. (E15)*

### Choosing to Let Go

Despite the meanings attributed to sexual health, in an expanded way, based on social interactions with the postpartum period, women determine their course of action and openly prioritize caring for their child and husband over caring for themselves. In interpreting the actions of others, the participants perceive a resistance to collaboration and division of tasks by their partners. As such, they interpret their actions as giving them greater responsibility for caring for the newborn and looking after the house. As a result, they feel overloaded, exhausted, worn out and consequently afraid that they won’t be able to cope.

*Sometimes it’s like I tell you, the tiredness, that sometimes we’re always taking care of ourselves and leaving ourselves last, I myself leave myself last a lot (E15).*

*I let myself go, because, without realizing it, I ended up living as a function of him, as a function of my son, always looking out for his well-being, how he’s doing, if he’s eaten, if he’s slept well, and I didn’t look at myself. (E8)*

*If he helped me more, I think I’d be less burdened, I think we’d have a healthier sex life, something that wasn’t tiring, which is what it is for me. (E18)*

In love relationships, in a context of pressure and self-blame, prioritizing others, women act by having sex even if they don’t want to. The reasons for acting in this way are related to the fact that they are married, afraid of the relationship “cooling off”, afraid of their husband looking for someone else, and insecurity. Thus, women prioritize their partner’s satisfaction and care less about their own pleasure. Another important point is the pressure from their partners to return to sexual activity. Women interpret this as meaning that they “owe” or that they have no desire. This leads to negative feelings such as abuse and guilt.

*He kind of jokes that I owe him, so I realize I have to show up. (E3)*

*You’re doing something you don’t want to do because you want to please someone else. It’s a feeling of abuse, as if you were being violated, abused. There are days when I wake up wanting to cry, because I did something I didn’t want to do and my space wasn’t respected at that moment and in this phase I’m going through. Because I thought the problem was with me, I used to say how come I’m so young and I don’t feel like having sex at all. (E18)*

*I started to feel like it was an obligation that I had to have because I’m married and my husband needs it. (E18)*

*We’re a bit afraid, aren’t we, because if you’re missing and he ends up looking for you on the street. (E3)*

*More because I’m really insecure, because I think, damn, my boyfriend wants to have sex and I don’t want to, I’m insecure about my body, insecure about motherhood and insecure about my relationship. (E14)*

### Reframing the Postpartum Period to Experience Healthy Sexual Health

By reviewing perspectives, establishing the situation and defining a course of action, regardless of the expectations surrounding the postpartum period, the participants recognize that it is a period made up of various changes in life and sexual health, which require adaptations and adjustments. They report that, after a period of time, they tend to adapt better to these new realities.

*It’s just that today we’re better able to deal with things, isn’t it? We’ve got used to the baby, his routines, we have more time to ourselves, so it’s more relaxed.” (E1)*

To this end, the participants emphasize that, as coping strategies, more complete information and open dialogue, from the family to the professional sphere, can minimize the problems surrounding postpartum sexuality and effectively promote sexual health. In this context, they claim that health professionals don’t provide information spontaneously, only when asked. As a result, women don’t feel open about their doubts.

*They don’t touch on the subject, neither of the two doctors did. Except when I asked if I could have sex with my husband. (E20)*

*They could have spoken, opened up, couldn’t they? If they had said: “Do you have any doubts?”, “Do you have any questions?”, something like that. But they don’t ask, they don’t open up, so we’re left feeling a bit like this.” (E3)*

*There should be an approach, from within the home, from our mother, our family. When we get out there, we’re already more open-minded, we already know more.” (E2)*

As another strategy, the participants consider that a consolidated support network can have a positive impact on sexual health in the postpartum period, making it possible to share responsibilities and care for the home and children, reducing the burden on women and helping them to be more disposed to sexual health. And, in terms of the professional support network, they would have liked to have had access to quality information on sexual health, with effective participation and space to discuss sexual health.

*So, the more you have someone to help you in your day-to-day life, the more rested you’ll be, you’ll have more energy, you’ll be more active. (E16)*

*I think that when you have a support network, so you don’t get so overwhelmed, things flow. (E9)*

*So, I wish I’d been guided so that, when this phase came, I wouldn’t have blamed myself as much as I did. I’d have known how to deal with it more and it would have helped with sexual relations, I’d have felt more comfortable with him. (E18)*

Through the interpretative process, meanings are manipulated and can be modified. Thus, a process of reframing the postpartum period was observed by recognizing the importance of taking care of oneself in biopsychosocial aspects in order to care for others. Despite the challenges encountered in the postpartum period and the lack of support, they define as a line of action the attempt to recover self-care, including beauty care and mental health care, in order to achieve a healthy sexual experience and in accordance with the *meanings attributed to sexual health.*

*I went back to my routine of looking after my skin, cleaning it, applying foundation, concealer, fixing it up, because, like it or not, it helps my self-esteem, and then I felt alive again. (E16)*

*I’m trying to look a bit more at my mental health, which I think will have an impact on all the others, both physical, sexual and social. (E6)*

### Representative Model

**Figure 1:**
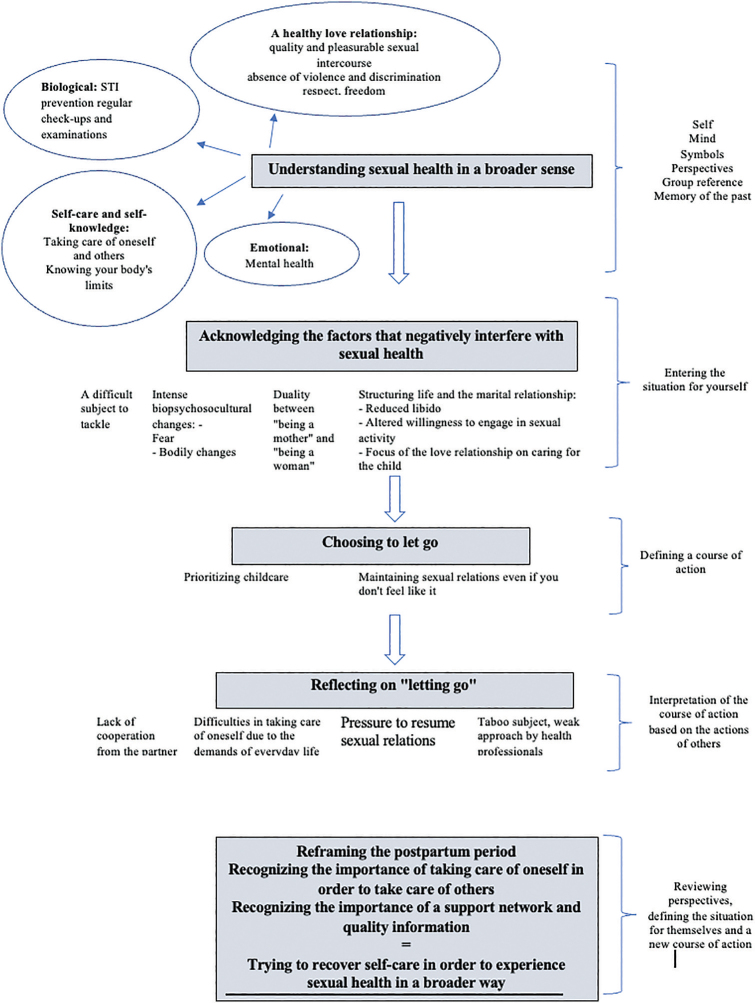
Schematic representation of puerperal women’s social interaction with their sexual health.

Source: Author.

Based on the results obtained, a representative diagram (Figure 1) was constructed of the process of social interaction between puerperal women and their sexual health, based on the meanings attributed by them, in other words, an explanatory model from an interactionist perspective, based on the scheme proposed by Charon^(10)^.

## DISCUSSION

Based on the constant interaction with their close social group, the participants in this study meant sexual health to go beyond sex and an active sex life, meeting the concept of sexual health as the exercise of sexuality with physical, emotional, mental and social well-being, encompassing not only aspects related to reproductive health, but also the possibility of having safe and pleasurable sexual experiences, without coercion, discrimination and violence^(18,19)^.

Sexual health is a symbolic social object, and its meanings are expressed in society as a taboo subject that is difficult to approach^(20)^, thus often remaining invisible, particularly in the postpartum context, where it is socially constructed that women should be focused on motherhood and little is said about this subject^(21,22)^. From an interactionist perspective, although there is no single way of explaining situations^(10,11)^, this invisibility is a significant symbol, in which the various changes in the postpartum period make it a period susceptible to disorder and emotional imbalance, influencing the self-esteem and self-image of postpartum women^(23)^.

Image disturbance is a factor that directly influences the meanings attributed to women and, consequently, how they act in relation to sexual health. It is worth highlighting the meanings attributed to breasts, which were previously a symbol of femininity and sensuality and, in the postpartum period, as a “feeding machine” for the baby with almost no sensitivity^(24)^, which can make them feel less attractive, reduce vaginal lubrication and libido^(8)^. This situation is exacerbated by the socially constructed aesthetic standard based on social interactions, mainly through the media, and by changes in the marital relationship after the birth of a child^(25,26)^.

It is clear that the social roles attributed to women and men do not have the same social recognition, strengthening the hierarchical inequality of female subordination in the face of male hegemony^(26)^. According to the maternal role, women are expected to take on most of the responsibility for household chores and caring for the newborn, as was pointed out in this study, in which, as a social action, women spend most of their time caring for the baby, causing a feeling of loss of femininity and longing for their personal space, now lost^(7,21)^.

In the context of pressure, lack of cooperation from the partner and difficulty in taking care of oneself, gender issues, patriarchy and the reproduction of social roles play crucial roles in the experience of puerperal women with regard to their sexual health and sexual pleasure. These complex elements can lead to the repression of female sexuality, where women may feel inhibited in exploring their own sexuality, and even learning about their own bodies and desires^(20)^. However, it is essential to raise awareness, strengthen and train health professionals in a comprehensive and discrimination-free approach. In this way, women can have access to comprehensive, quality information on sexual health, so that they can make informed decisions, promoting the search for more equitable and satisfying sexual relationships. In other words, the promotion of gender equality and quality sex education are fundamental in empowering women to live healthy, full and satisfying sex lives^(21)^.

Social interactions with the family in the postpartum period are marked by imbalances in love relationships, difficulties in the division of tasks between the couple and difficulties in adapting to new demands^(25)^. Thus, women act out, striving to meet the needs of the newborn and other family members, leading to fatigue, overload, anxiety and decreased self-care, neglecting themselves and, consequently, experiencing challenging postpartum sexual health^(7)^.

Corroborating the results of this study, other studies have shown that feelings of self-blame and concern about having sex, even without wanting to, were present. They described their return to sexual activities as motivated by pleasing their partners and characterized their return to sexual activities as complicated and frightening, because they feared the unknown, including physical pain during sexual intercourse and fear of a new pregnancy^(5–7)^.

Considering that individuals are able to use their mental resources, background and symbolization skills to interpret and adapt reflexively to situations as they themselves define them^(10,11)^, it is considered essential to prepare women and their partners for the changes of the postpartum period from prenatal onwards, as a possible reducer of stress, anxiety and fear^(23)^, in other words, meanings are manipulated and can be modified^(10,11)^. However, they feel that there is little discussion of the subject in health care, that they are not questioned about it, that their attention is focused on the baby, and that they are dissatisfied with the information they receive, generating a feeling of neglect^(6,7)^.

It’s worth noting that women miss talking openly about their sexual health in the puerperium, including the negative aspects and difficulties^(6)^. Despite the challenges, frequent consultations with health professionals during the pregnancy-puerperium cycle are opportune spaces for approaching and recommending safe postpartum sexual relations and counseling^(5,27)^. Skilled nursing care based on the prevention of complications, physical and emotional comfort and health education are essential^(22)^. Thus, health professionals can be involved in this context to contribute to the process of reframing the postpartum period in order to experience healthy sexual health.

In addition, a consolidated support network has a significant impact on women’s social interactions during the pregnancy-puerperium cycle, since it not only helps with the care of the newborn and the puerperal woman herself, but also promotes a sense of strength and improved self-esteem. Prenatal care is an opportunity to investigate and strengthen this network^(22)^. Therefore, with qualified information about their bodies and their health, women can start to have greater decision-making and planning power over their lives^(19,21)^.

The study’s limitations include the fact that, although data collection via videoconference allows for greater participation by people from different locations, it excludes women who do not have access to the internet, such as those in the worst socio-economic situations. Furthermore, the interviewees who volunteered were living in heterosexual relationships, making it impossible to say that the results achieved can be generalized to all types of relationships.

## CONCLUSION

The meanings attributed by the puerperal women to sexual health after childbirth are in the sense of comprehensive health care. However, based on social interactions in the duality between “being a woman” and “being a mother” in the postpartum period, they recognize fear, bodily transformations and changes in focus from the love relationship to caring for the baby as intervening factors in their sexual health, and imply the social actions of these women. In the context of dynamic social experience, puerperae actively adapt to circumstances and choose to put themselves aside, prioritizing the care of others. In this interactional process, they re-signify the postpartum period by recognizing the importance of taking care of themselves in biopsychosocial aspects and try to rescue self-care for a healthy sexual experience.

We believe that the results of this study can contribute to raising awareness and training health professionals in the context of continuing education. In this way, the production of scientific knowledge should be expanded and strategies for translating knowledge should be created to support the inclusion of this theme in educational actions and care plans for women, both in prenatal and postpartum consultations. In this way, the aim is to meet the demands and help ensure that women’s social interaction with their sexual health actually involves biopsychosocial aspects and promotes healthy experiences.
